# ALDH_2_ knockout protects against aortic dissection

**DOI:** 10.1186/s12872-022-02874-5

**Published:** 2022-10-13

**Authors:** Chentao Luo, Bing Zhou, Yong Cui, Zhifang Liu, Shuwei Wang

**Affiliations:** Heart Center, Department of Cardiovascular Surgery, Zhejiang Provincial People’s Hospital (Affiliated People’s Hospital, Hangzhou Medical College), 310014 Hangzhou, Zhejiang China

**Keywords:** Aorta dissection, ALDH_2_ knockout, β-aminopropionitrile

## Abstract

**Background:**

The incidence and mortality of aortic dissection (AD) are increasing. In pathological studies, macrophages, T lymphocytes and dendritic cells were found in the tunica media of the aorta. Acetaldehyde dehydrogenase 2 (ALDH_2_) gene polymorphisms are associated with a high incidence of hypertension in Asian populations. However, there is no clear evidence of the relationship between ALDH_2_ and aortic dissection in Asians. The aim of this study was to investigate the incidence of aortic dissection in different ALDH_2_ genotypes and explore changes in the vasculature.

**Materials and methods:**

Three-week-old male mice were administered freshly prepared β-aminopropionitrile solution dissolved in drinking water (1 g/kg/d) for 28 days to induce TAD. An animal ultrasound imaging system was used to observe the formation of arterial dissection and changes in cardiac function. Subsequently, mice were euthanized by cervical dislocation. The aortas were fixed for HE staining and EVG staining to observe aortic elastic fiber tears and pseudoluma formation under a microscope.

**Results:**

Knockout of ALDH_2_ mitigated β-aminopropionitrile-induced TAD formation in animal studies. Ultrasound results showed that ALDH_2_ knockout reduced the degree of ascending aortic widening and the incidence of aortic dissection rupture. Pathological sections of multiple aortic segments showed that the protective effect of ALDH_2_ knockout was observed in not only the ascending aorta but also the aortic arch and descending aorta. The expression levels of genes related to NK CD56bright cells, Th17 cells, T cells and T helper cells were decreased in ALDH_2_ knockout mice treated with β-aminopropionitrile for 28 days.

**Conclusion:**

ALDH_2_ knockout protects against aortic dissection by altering the inflammatory response and immune response and protecting elastic fibers.

## Introduction

Aortic dissection (AD) is one of the most dangerous aortic diseases [1]. Aortic rupture or aortic intramural hematoma causes AD [2]. The false lumen of a dissection influences the blood supply from the heart to other organs (brain, kidneys, mesentery, etc.) [3]. It has been reported that the 24-hour mortality rate of AD is approximately 30 to 40% [4]. Aortic disruption or pericardial tamponade could lead to acute death [5]. AD usually occurs in the elderly population and has an incidence rate of 40/100,000 per year [6]. However, recent clinical work has found that patients with AD are becoming increasingly younger [7]. Hypertension, arteriosclerosis, Marfan syndrome and congenital bilobar aortic valve malformation are common causes of AD [8]. It has been reported that WBC (white blood cell) infiltration, vascular inflammation and extracellular matrix remodeling are the main pathogenic factors of AD [9]. In the process of aortic remodeling and dilatation, the destroyed integrity of the tunica media of the aorta leads to the occurrence of this disease [10]. Angiotensin 2 is the main effector peptide of the renin-angiotensin-aldosterone system [11]. It can constrict blood vessels to raise blood pressure. It has been reported that hypertrophy of vascular smooth muscle cells and infiltration of inflammatory cells damage blood vessels. This is one of the reasons why hypertensive patients are prone to AD.

Acetaldehyde dehydrogenase 2 (ALDH_2_) is one of the key enzymes for metabolizing alcohol and aldehydes [12]. The ALDH_2_ gene consists of 13 exons, and the Glu504Lys polymorphic locus (rs671) located at the 12th position is widely present in East Asian populations [13]. The mutation rate at this locus is as high as 40%, and it has been reported that ALDH_2_ protects heart muscle cells and nerve cells [14]. However, to date, research on the effect of ALDH_2_ on AD and its underlying mechanisms is still in the exploratory phase.

In this study, we demonstrated that ALDH_2_ knockout (KO) is a protective against AD. Interventions targeting the ALDH_2_ gene may become a new treatment option for the prevention of TAD.

## Materials and methods

### Mice and models

ALDH_2_ KO mice were obtained from Fudan University. Three-week-old wild-type (WT) C57BL/10 mice were purchased from Changzhou Cavens Laboratory Animal Co. Ltd. Mice were housed in the animal facility of Zhejiang Provincial People’s Hospital. β-aminopropionitrile (BAPN) treatment led to AD formation by inhibiting lysyl oxidase activity [15]. Three-week-old male WT C57BL/10 mice were fed a normal diet and administered freshly prepared BAPN (Sigma‒Aldrich, St. Louis, MO, USA) solution dissolved in drinking water (1 g/kg/d) for 28 days in the experimental group (n = 12). Three-week-old male ALDH_2_ KO mice were fed a normal diet and administered freshly prepared drinking water for 28 days as previously described for the control group (n = 12). In the process of feeding with different reagents (BAPN or water), some of the mice (n = 8) died suddenly, and their aorta and blood samples were preserved within two hours. The surviving mice (n = 40) were euthanized by cervical dislocation for further animal experiments. All protocols were approved by the Institutional Animal Care and Use Committee of Zhejiang Provincial People’s Hospital (No. 2021QT200).

### Total RNA extraction

Total RNA was extracted from the tissues using TRIzol (Invitrogen, Carlsbad, CA, USA) according to the manufacturer’s instructions. Approximately 60 mg of tissue was ground into powder by liquid nitrogen in a 2 mL tube, homogenized for 2 min and rested horizontally for 5 min. The mix was centrifuged for 5 min at 12,000 × g at 4 °C, and then the supernatant was transferred into a new EP tube with 0.3 mL chloroform/isoamyl alcohol (24:1). The mix was shaken vigorously for 15 s and then centrifuged at 12,000 × g for 10 min at 4 °C. After centrifugation, the upper aqueous phase, where the RNA remained, was transferred into a new tube with an equal volume of isopropyl alcohol and then centrifuged at 13,600 rpm for 20 min at 4 °C. After discarding the supernatant, the RNA pellet was washed twice with 1 mL 75% ethanol, and then the mix was centrifuged at 13,600 rpm for 3 min at 4 °C to collect residual ethanol. This was followed by air drying for 5–10 min in the biosafety cabinet. Finally, 25 µL ~ 100 µL of DEPC-treated water was added to dissolve the RNA. Subsequently, the total RNA was qualified and quantified using a Nano Drop and Agilent 2100 bioanalyzer (Thermo Fisher Scientific, MA, USA).

### mRNA library

Construction Oligo(dT)-attached magnetic beads were used to purify mRNA. Purified mRNA was fragmented into small pieces with fragment buffer at the appropriate temperature. Then, first-strand cDNA was generated using random hexamer-primed reverse transcription, followed by second-strand cDNA synthesis. Afterward, A-Tailing Mix and RNA Index Adapters were added, and this mixture was incubated to perform end repair. The cDNA fragments obtained from the previous step were amplified by PCR. The products were purified by Ampure XP Beads and then dissolved in EB solution. The product was validated on an Agilent Technologies 2100 bioanalyzer for quality control. The double-stranded PCR products from the previous step were heated, denatured and circularized by the splint oligo sequence to obtain the final library. The single strand circular DNA (ssCir DNA) was formatted as the final library. The final library was amplified with phi29 to make DNA nanoballs (DNBs), which had more than 300 copies of one molecule. DNBs were loaded into the patterned nanoarray, and paired-end 100-base reads were generated on the BGIseq500 platform (BGI-Shenzhen, China).

### Aortic ultrasound

We used a high-resolution microultrasound system (Vevo 2100; VisualSonics) equipped with a 30-MHz transducer. This procedure was performed on live mice in all groups once every three days to observe the heart and aorta, and we matched the last data of mice that suffered from acute death with the endpoint events (dissection rupture death, dissection survival or no dissection formation). To perform ultrasound scans, mouse hair was removed from the chest, and the mice were anesthetized with 1% isoflurane. Mice were laid supine, and the probe was placed at the right edge of the sternums of the mice. The probe was 30 ~ 45-degree angles from the chest walls of the mice. The ascending aortas, aortic arches and their branches were observed. Longitudinal images of the aortas were obtained in B-mode, and the internal diameter of the aorta was measured.

### Histology and immunohistochemistry

The aortas were harvested, fixed in 4% paraformaldehyde, embedded and sectioned at 4-µm intervals. Aortas were stained with hematoxylin and eosin for histological evaluation. Pathological changes in the aortas were observed under a microscope. The tissues were stained by elastin staining immunohistochemistry, and the positive rate was calculated. After the section was dewaxed, the tissue was stained with Verhoeffs van Gieson to observe the changes in elastic fibers and collagen fibers.

### TUNEL

The TUNEL assay was performed in the following consecutive steps. First, after reheating, the frozen sections were fixed with cold acetone for 10 min and washed on a shaker three times in PBS for 5 min each time. Second, protease K (Servicebio) working fluid was added to cover the tissue and was incubated at 37 °C for 25 min. The slides were then placed in PBS (pH 7.4) and washed on a decolorizing shaker for 30 min. Third, the appropriate amount of reagent 1 (TdT) and reagent 2 (dUTP) from the TUNEL kit (11,684,817,910, Roche) was mixed into the reaction solution at a ratio of 1:9, and the sections were placed in the reaction solution for incubation at 37 °C for 2 h. Fourth, the sections were placed in 3% hydrogen peroxide solution prepared with methanol (hydrogen peroxide:methanol = 1:9) and incubated in the dark for 15 min at room temperature. Fifth, after washing with PBS, the appropriate amount of reagent 3 (converter-POD) was added to cover the tissue. The sections were placed flat in a wet box and incubated at 37 °C for 30 min. Sixth, after washing with PBS, the freshly prepared DAB was added to the glass slides for color development. Seventh, Harris hematoxylin was used to redye the tissue for 3 min, and then the samples were dehydrated and sealed.

ALDH_2_ Genotype.

The ALDH_2_ gene is located on chromosome 12 and contains 13 exons. The rs671 mutation of the ALDH_2_ gene is located in exon 12 at position 1510, and guanine (G) is mutated to adenine (A). There are three common combinations in the population: GG (wild homozygous), GA (mutant heterozygous), and AA (mutant homozygous). The restriction endonuclease could recognize and cut off the specific base sequences of the DNA double strands. We amplified the target fragment by PCR and digested it with the corresponding restriction nuclear acid digestion enzyme. Because of the different base compositions, different lengths of enzyme digestion fragments can be produced. SNP typing was performed by capillary electrophoresis. The enzyme used was TspRI.

### Statistical analysis

All experiments were conducted 3 times. The results are presented as the mean ± standard error of the mean (SEM). The data were evaluated by one-way analysis of variance (ANOVA) followed by Dunn’s test using GraphPad Prism software. P values < 0.05 were considered statistically significant. The sequencing data were filtered with SOAPnuke (v1.5.2) by (1) removing reads containing sequencing adapters, (2) removing reads whose low-quality base ratio (base quality less than or equal to 5) was more than 20%, and (3) removing reads whose unknown base (‘N’ base) ratio was more than 5%. Afterward, clean reads were obtained and stored in FASTQ format. The clean reads were mapped to the reference genome using HISAT2 (v2.0.4). After that, Ericscript (v0.5.5) and rMATS (v3.2.5) were used to fuse genes and differentially spliced genes (DSGs), respectively. Bowtie2 (v2.2.5) was applied to align the clean reads to the gene set. A database for this organism was built by BGI (Beijing Genomic Institute in ShenZhen), which includes known and novel coding transcripts. KEGG (Kyoto Encyclopedia of Genes and Genomes) is a knowledge base for systematic analysis of gene functions, linking genomic information with higher order functional information [16]. Kanehisa laboratories has provided a KEGG Business License to BGI. Then, the expression level of the gene was calculated by RSEM (v1.2.12). The heatmap was drawn by pheatmap (v1.0.8) according to the gene expression levels in different samples. Essentially, differential expression analysis was performed using DESeq2 (v1.4.5) with a Q value ≤ 0.05. To gain insight into the change in phenotype, GO and KEGG enrichment analyses of annotated differentially expressed genes were performed by Phyper based on the hypergeometric test. The significance levels of terms and pathways were corrected by the Q value with a rigorous threshold (Q value ≤ 0.05) by Bonferroni.

## Results

### Knockout of ALDH_2_ mitigated BAPN-induced AD formation

**Fig. 1:**
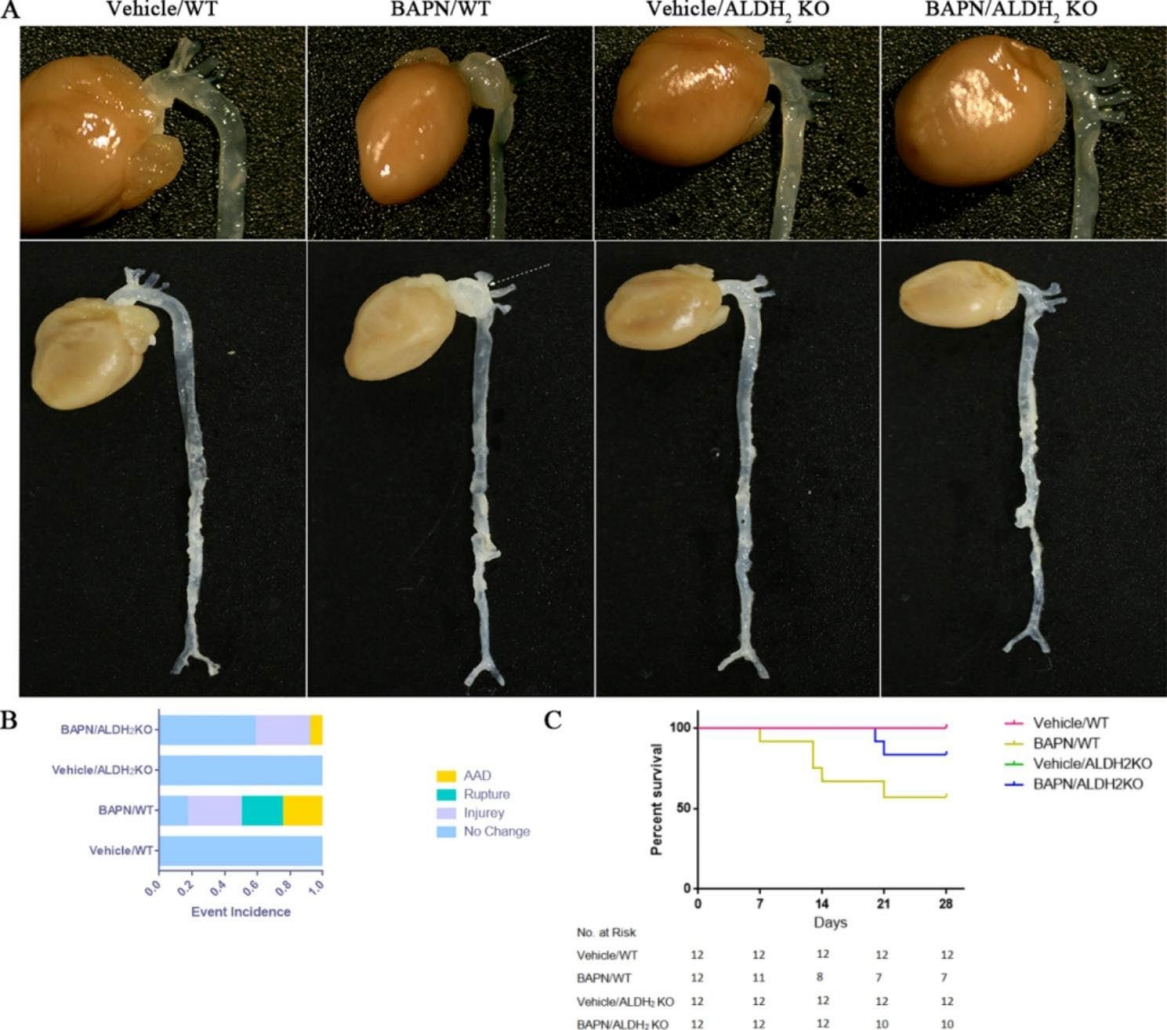
(A) Representative images of whole aortas from either vehicle- or BAPN-treated WT and ALDH_2_ KO mice. (B) Bar graph showing the incidence of TAD formation and rupture in either vehicle- or BAPN-treated WT and ALDH_2_ KO mice (n = 12). (C) Survival analysis in either vehicle- or BAPN-treated WT and ALDH_2_ KO mice. (n = 12)

To determine the role of ALDH_2_ in AD, we injected ALDH_2_ KO and WT mice with either vehicle or BAPN. Gross examination revealed that the vascular tissue of WT mice had no obvious change after 1 month of vehicle administration. The vascular tissue of WT mice showed obvious changes after 1 month of BAPN administration. The ascending aorta and aortic arch in the BAPN/WT group were dilated, and the blood vessel wall was thinner than that in the other groups. Typical AD could be recognized after 1 month of BAPN administration (Fig. 1 A). The vascular tissue of ALDH_2_ KO mice showed no obvious change after 1 month of vehicle and BAPN administration. The bar graph shows the incidence of TAD formation or rupture in WT and ALDH_2_ KO mice treated with either vehicle or BAPN. After treatment with BAPN, WT mice exhibited higher levels of TAD formation compared with ALDH_2_ KO mice (Fig. 1B). Kaplan‒Meier survival curves showed that ALDH_2_ knockout mitigated BAPN-induced TAD formation (Fig. 1 C).

### ALDH_2_ knockout reduces the degree of aortic dilation and the rate of ascending aortic aneurysm or dissection

**Fig. 2:**
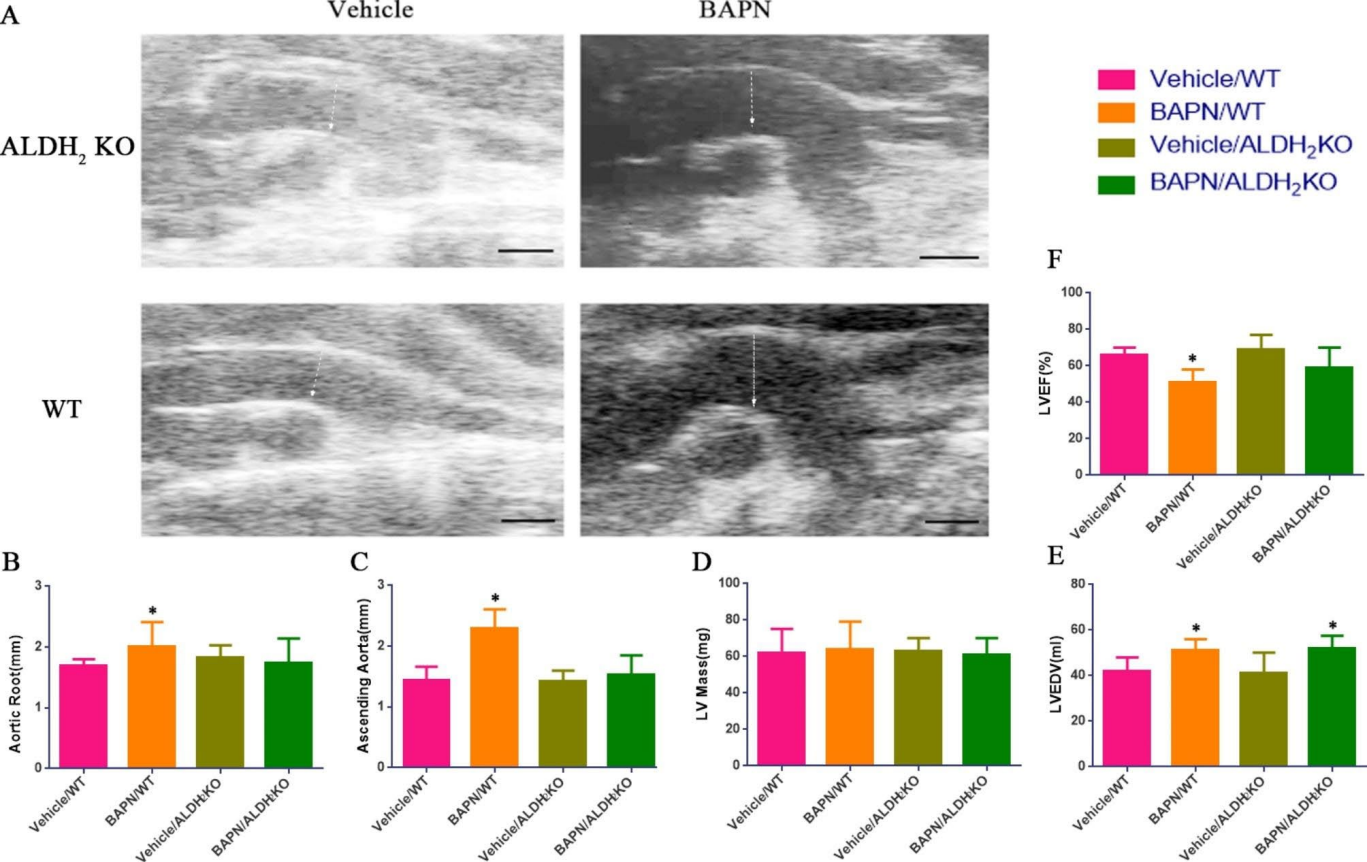
(A) Representative ultrasound images of aortas (white arrows) from in WT and ALDH_2_ KO mice treated with either vehicle or BAPN. Scale bar: 1 mm. (B, C, D, E, F) Statistical analysis of ultrasound measurements of WT and ALDH_2_ KO mice treated with either vehicle or BAPN (n = 12)

A BAPN administration-induced TAD model was made. Representative ultrasound images of the aortas (white arrows) from WT and ALDH_2_ KO mice treated with either vehicle or BAPN for one month are shown in Fig. 2 A (scale bar: 1 mm). The ultrasound results showed that the diameters of the aortic root and ascending aorta in the ALDH_2_ KO group treated with BAPN for one month were not significantly different from those in the WT group treated with vehicle. The aortic root and ascending aorta in the WT group treated with BAPN for one month were significantly more dilated, which was related to the high rate of aortic aneurysm or aortic dissection in this group (Fig. 2B, C). The mass of the left ventricle of the mice in the four groups was not significantly different (Fig. 2D). This may be related to the acute attack of this disease. The left ventricular end diastolic volume (LVEDV) of mice treated with BAPN was larger than that of mice treated with vehicle (Fig. 2E). The increased pressure load resulted in the enlargement of the LVEDV. The left ventricular ejection fraction (LVEF) of WT mice in the group treated with BAPN was lower than that in the other groups (Fig. 2 F).

### ALDH_2_ knockout protected the elastic fibers of aortas treated with BAPN for one month

**Fig. 3:**
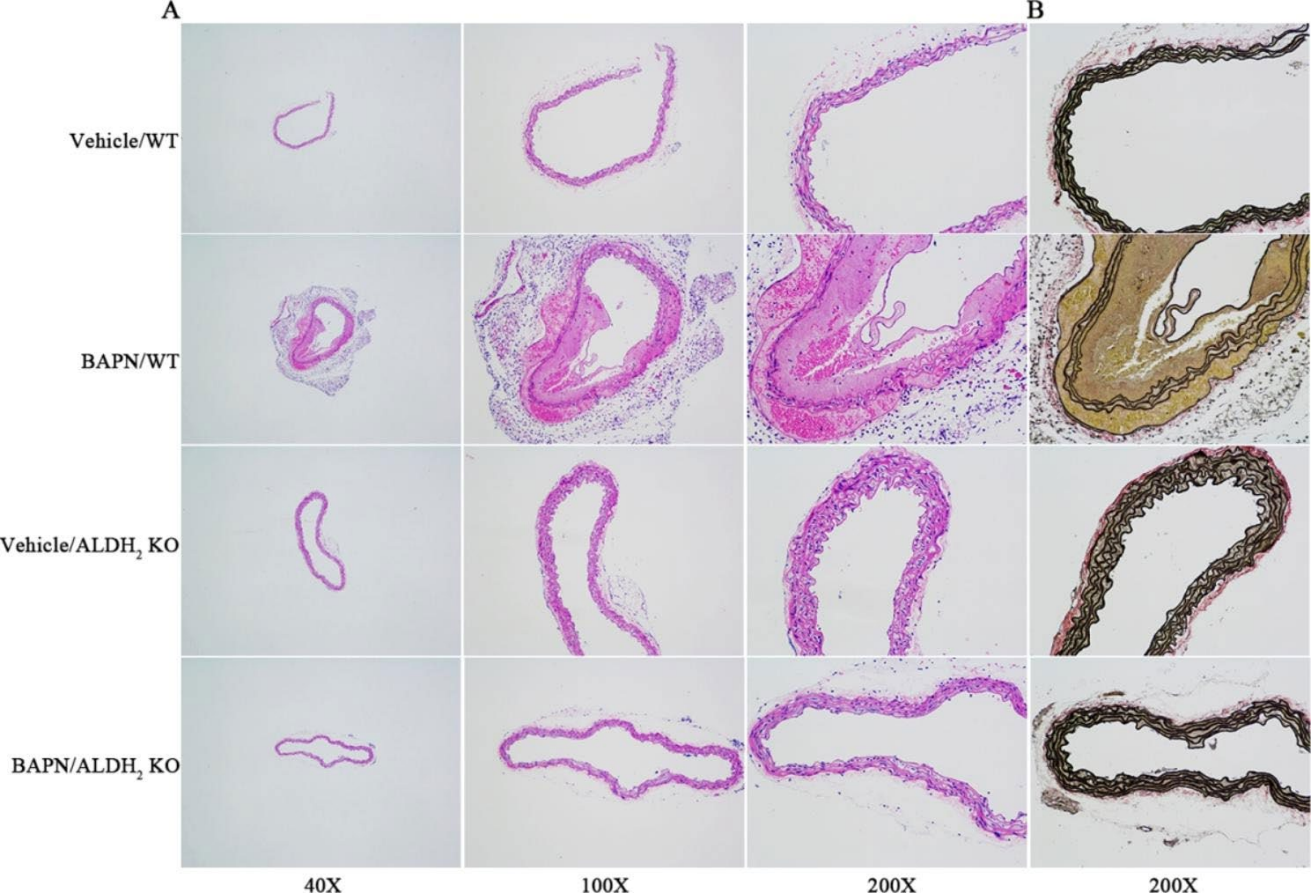
(A) Representative images of HE staining in either vehicle- or BAPN-treated WT and ALDH_2_ KO mice. (B) Representative images of EVG staining in either vehicle- or BAPN-treated WT and ALDH_2_ KO mice

Hematoxylin and eosin and elastin staining revealed that the vascular elastic fiber of wild-type mice had no obvious change after 1 month of vehicle administration (Fig. 3 A). The vascular elastic fiber of WT mice had obvious changes after 1 month of BAPN administration. Severe elastic fiber fragmentation and mild dissection could be recognized after 1 month of BAPN administration. The vascular elastic fiber of ALDH_2_ KO mice showed no obvious change after 1 month of vehicle and BAPN administration. Representative images showed that ALDH_2_ deficiency suppressed BAPN-induced elastin degradation in mouse aortas (Fig. 3B).

### ALDH_2_ knockout reduced the aortic vascular injury caused by BAPN treatment

**Fig. 4:**
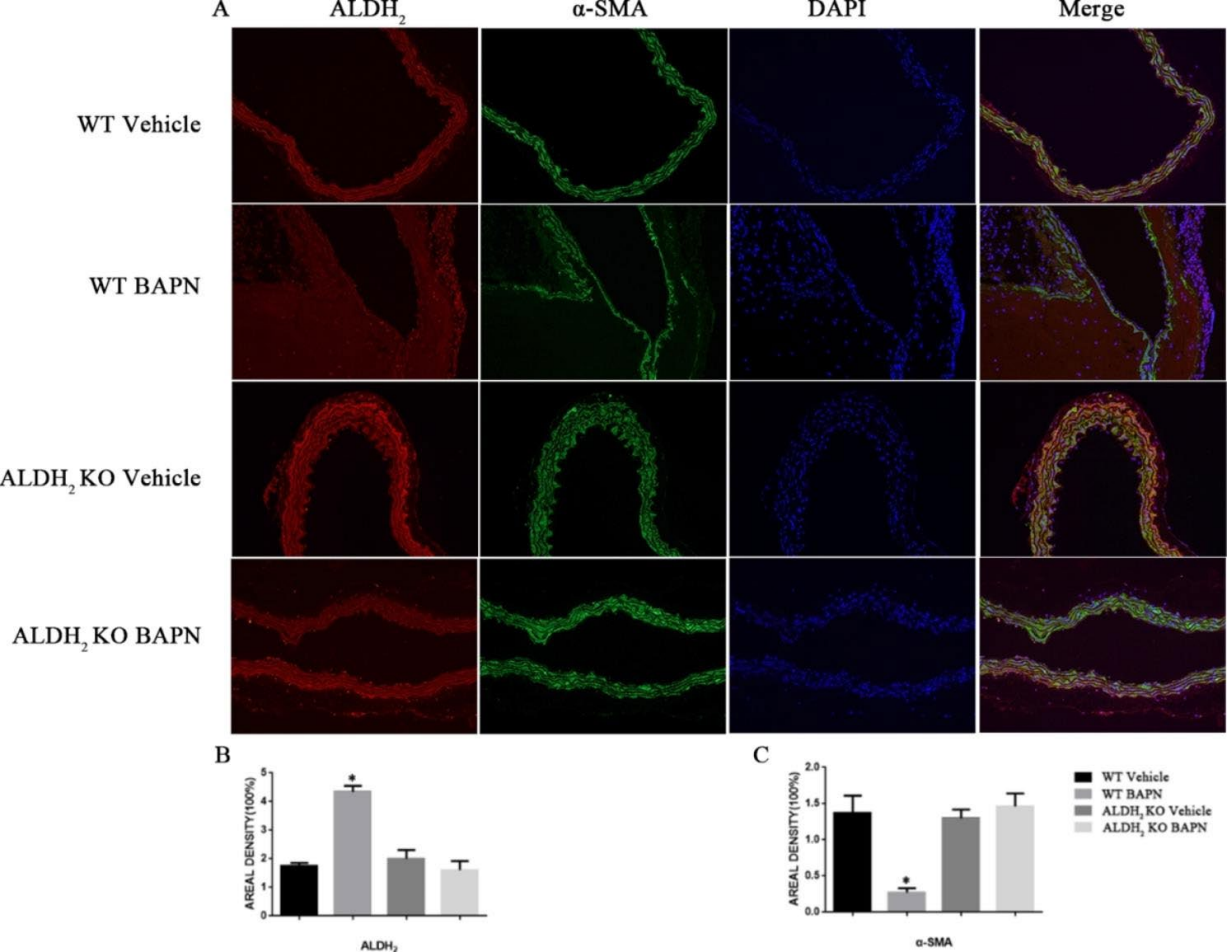
ALDH_2_ and α-SMA production in mouse aortas. (A) Representative immunofluorescence images of ALDH_2_ and α-SMA levels (n = 6). Scale bar: 200 μm. (B, C) Statistical analysis of ALDH_2_ and α-SMA levels (*p < 0.05, versus GG-Normal).

Immunofluorescence of ALDH_2_ and α-SMA was performed on four groups of samples (Fig. 4 A). The results showed that the aortas of mice in the WT group formed obvious damage and separations one month after treatment with BAPN. The expression level of ALDH_2_ was significantly higher in the BAPN-treated WT group than in the vehicle-treated WT group (Fig. 4B). The expression level of α-SMA was significantly lower in the BAPN-treated WT group than in the other three groups (Fig. 4 C). In the WT group treated with BAPN, the tunica media of the aorta were discontinuous, and various nuclei were gathered in the pseudolumen. In the other three groups, the intima media and adventitia of the aorta were continuous without obvious dissection.

### ALDH_2_ knockout protected the aortic root, ascending aorta, and aortic arch against BAPN treatment for one month and even affected descending aortic dilation

**Fig. 5:**
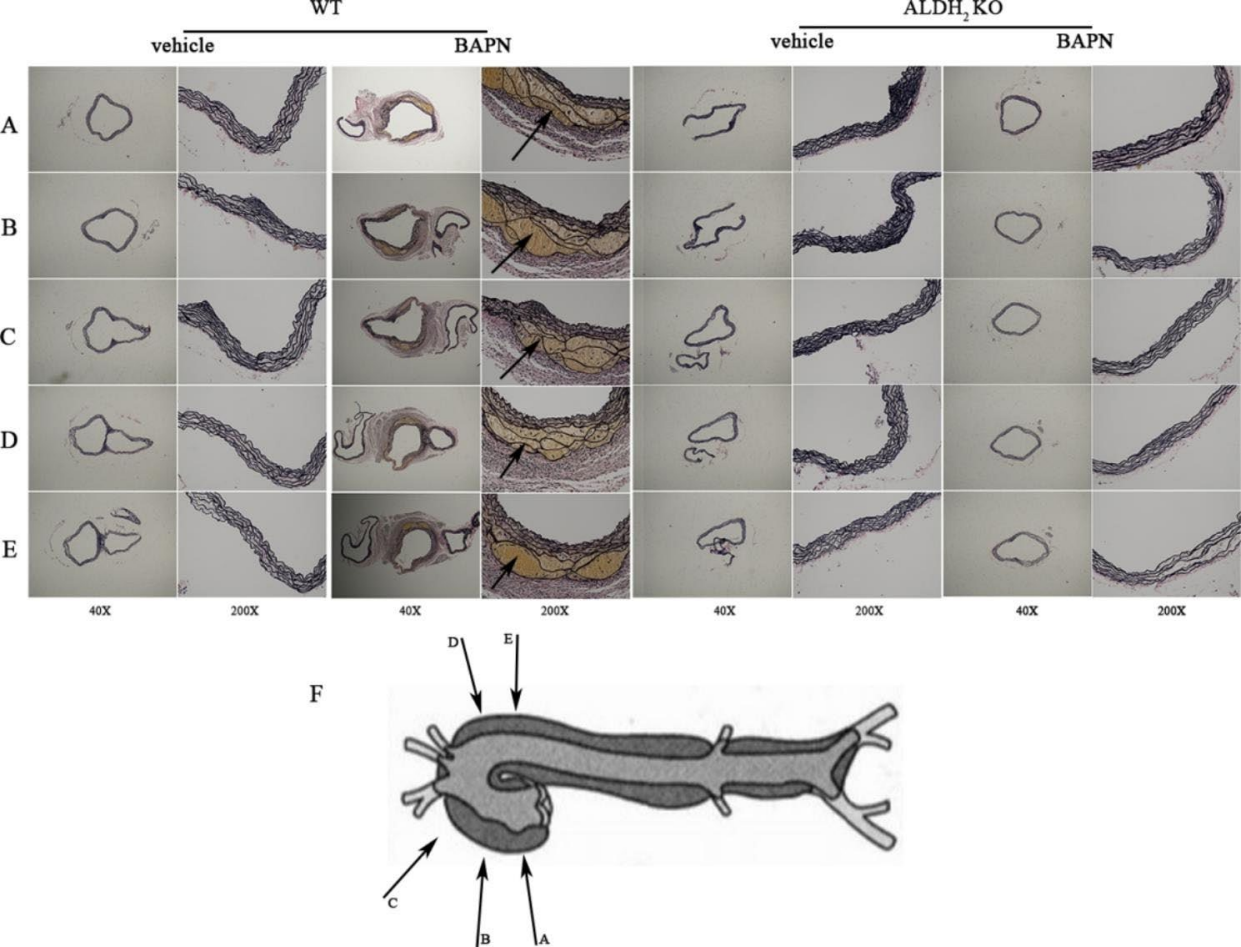
ALDH_2_ knockout mice are resistant to TAD. (A, B, C, D, E) Representative images of whole aortas and EVG staining images from mice treated with either vehicle or BAPN (n = 6). Scale bar: 40, 200 μm. (F) Different section positions of each image

The measurement of the outer circumference of aortas on pathological cross sections was provided, and the outer circumference of the aorta was smaller in ALDH_2_ knockout mice treated with BAPN than in the other groups (Fig. 5 A, B, C, D, E). The aortic adventitia was weaker, the tunica media was more discontinuous, and the pseudolumen of the wall was filled with red blood cells and macrophages. We selected cross-sectional sections of the aortic root, ascending aorta, aortic arch, descending aortic arch and descending aorta (Fig. 5 F). The results showed dissections in the aortic root, ascending aorta, aortic arch, descending aortic arch and descending aorta in WT mice treated with BAPN for 1 month. These characteristics were not found in ALDH_2_ KO mice treated with BAPN for 1 month. In the two vehicle groups, the results indicated that there was no significant lesion in the aortic wall at each site.

### Global gene expression analysis was performed in aortas from murine models (treated with vehicle or BAPN for 28 days)

**Fig. 6:**
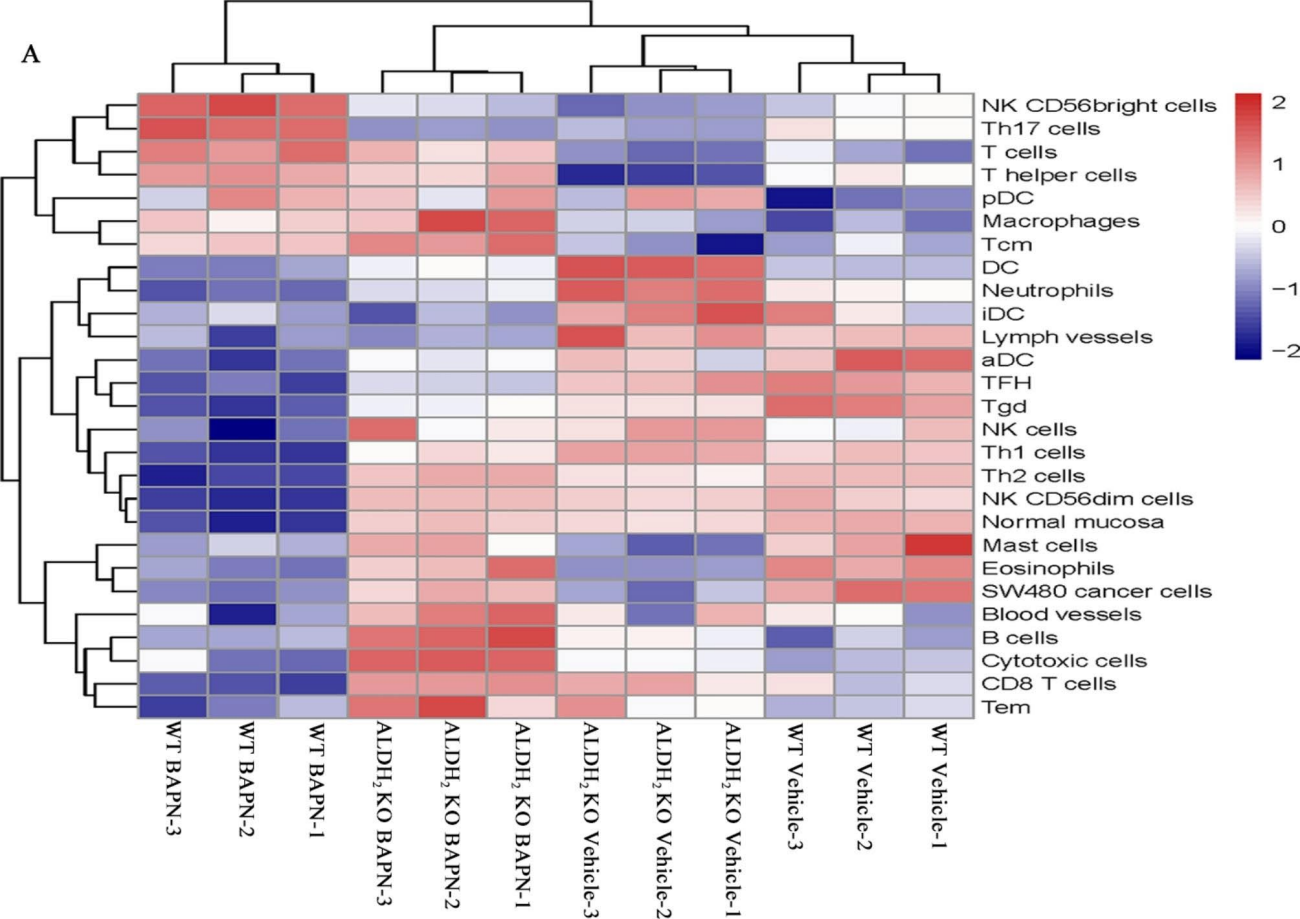
Global gene expression analysis was performed in aortas from murine models (BAPN induced for 28 days). A heatmap of the expression values is shown. Each point reveals the calculated log2 ratio of the indicated gene expression

A heatmap showed that the relative expression of genes related to blood vessels, B lymphocytes, cytotoxicity, CD8 T cells and macrophages was increased in ALDH_2_ knockout mice treated with BAPN at Day 28. The expression of genes related to NK CD56bright cells, Th17 cells, T cells and T helper cells was decreased in ALDH_2_ KO mice treated with BAPN at Day 28 (Fig. 6 A).

## Discussion

Aortic dissection is a serious disease of the aorta [17]. The intima of the aorta is impacted by blood flow, which causes a tear to form. The blood flows through the intima tear into the media to form a pseudolumen [18]. The pseudocavity is surrounded by a weak aortic adventitia, which can easily rupture [19]. Thrombosis in the pseudolumen is also one of the reasons for the poor prognosis of AD. During aortic replacement surgery, extracorporeal circulation is often established through femoral artery cannulation. Retrograde perfusion from the femoral artery may carry the thrombus from the rupture into the body. The thrombus flows through the bloodstream into the innominate artery, the left common carotid artery, the left subclavian artery, or other organs, leading to dysfunction of the corresponding organs [20]. All of the above effects lead to a high mortality and poor prognosis of AD.

The tunica media of the aorta is mainly composed of vascular smooth muscle cells, proteoglycans, mucopolysaccharides, elastic fibers and collagen fibers [21]. It has been proven that a decrease in vascular smooth muscle cells and injury to elastic fibers are the main causes of AD [22]. BAPN is an enzyme inhibitor that inhibits the cross-linking of collagen and elastin [23]. This inhibitor has been used to construct a C57BL/6J mouse model of AD [24].

A history of alcohol consumption has been reported as a risk factor for aortic dissection [25]. However, in clinical work, we found that the average age of patients with AD was decreasing, and an increasing number of patients had no history of habitual drinking. Studies have found that the ALDH_2_ GG type is one of the risk factors for the incidence of myocardial infarction in Japanese men [26]. We constructed AD models of WT and ALDH_2_ KO mice by BAPN treatment for one month. In the vehicle control group of WT mice, there was no AD or death. In the BAPN-treated group of WT mice, anatomy showed that approximately 41.7% of mice had formed aortic dilation or AD. A total of 25.0% of mice experienced sudden death, chest congestion or rupture. A total of 33.3% of mice had weak aortic walls and obvious injury, and only 16.7% of mice had normal aortas. Significant AD developed in 8.3% of the BAPN-treated ALDH2 KO mice. Intermural hematoma occurred in a few mice, which may be related to the rupture and hemorrhage of trophoblast vessels in the aortic adventitia. Active perivascular tissue dissection and active luminal tumor-like dilatation were found in 33.3% of this group. The vasculature was weaker and more transparent than normal aortic tissue, and multiple atherosclerotic plaques had formed in some locations. We performed echocardiograms once every three days to observe the heart and blood vessels of the modeled mice and matched the data and endpoint events (dissection rupture death, dissection survival or no dissection formation) of each mouse. The results indicated that the aortic root and ascending aorta of WT mice treated with BAPN were significantly widened compared with those in the vehicle control group. In ALDH_2_ KO mice, there was no significant difference in active vasculature widening between the two groups. It was found that the ventricular mass of the WT mice treated with BAPN increased at the same survival age, which might be related to the excessive cardiac pressure load during the growth and development of mice in this group. The results of HE and EVG staining indicated that the vascular lumen of WT and ALDH_2_ mice in the vehicle control group was intact. The tissue in the tunica media was continuous, and there was no intermural hematoma formation in the aortic adventitia. The vascular lumen of WT mice treated with BAPN showed the formation of a large pseudolumen, with a large number of red blood cells and macrophages in the pseudolumen. We indicated that the obstacle of true lumen blood supply, lumen thrombosis and rupture of pseudolumen might reduce the survival rate of mice. In ALDH_2_ KO mice treated with BAPN, the aortic pathological changes were similar to those in the control group, and the health of the aorta was better than that in the BAPN-treated WT group. The results of immunofluorescence colocalization experiments on frozen specimens at the same age showed that the expression level of α-SMA protein in the BAPN-treated WT group was significantly lower than that in the other groups. The pathological sections of this group conformed to the morphology of aortic dissection, and the smooth muscle cells of the vasculature were disorganized. The Stanford classification is divided into A and B types according to the needs of surgery [27]. For type A, the tear is located in the ascending aorta and is suitable for emergency surgery [28]. For type B, dissection lesions confined to the abdominal aorta or iliac artery can be treated medically, followed by endovascular treatment [29]. EVG staining was performed on the aortic root, ascending aorta, initiation of aortic arch, distal aortic arch and descending aorta of mice of the four groups. The results showed that BAPN-treated WT mice had a high incidence of Stanford type A aortic dissection. BAPN-treated ALDH_2_ mice did not suffer serious damage to the black elastic fibers and red collagen fibers in the thoracic and abdominal aorta. We suggest that ALDH_2_ KO may reduce the incidence of aortic dissection by protecting elastic fibers. We will focus on this in subsequent studies. Previous studies have focused on the role of metalloproteinases in AD. Our results demonstrated that NK CD56bright cells, Th17 cells, T cells and T helper cells influenced the formation of AD.

## Limitations

A limitation of the study is that the current results only involve male mice because the male to female prevalence of AD is 2.5:1. We will further investigate the differences in ALDH2 genotypes and incidence of AD between male and female mice. There were no short- or long-term clinical outcomes for patients with AD in this study. In the future, we will verify whether the results of this study are consistent with clinical results.

## Conclusion

ALDH_2_ knockout protected against AD development by altering the inflammatory response and immune response and protecting elastic fibers. We investigated the effects of ALDH_2_ knockout in a model of AD. The results also suggest that ALDH_2_ KO protects the ascending aorta, aortic arch and descending aorta. Further studies are needed to explore how inflammation and T cells are involved in this process.

## Data Availability

The dataset is available from the corresponding author on reasonable request.

## Abbreviations

*AD*: Aortic dissection.
*TAD*: Thoracic aortic dissection.
*ALDH*_*2*_: acetaldehyde dehydrogenase 2.
*BAPN*: β-aminopropionitrile.
*KO*: Knockout.
*WT*: Wild-type.
*GG*: Wild homozygous.
*GA*: Mutant heterozygous.
*AA*: Mutant homozygous.
*SMA*: Smooth muscle actin.
*LVEDV*: Left ventricular end diastolic volume.
*LVEF*: Left ventricular ejection fraction.
